# The Use of Different Components of Brain Oxygenation for the Assessment of Cerebral Haemodynamics: A Prospective Observational Study on COVID-19 Patients

**DOI:** 10.3389/fneur.2021.735469

**Published:** 2021-12-20

**Authors:** Chiara Robba, Danilo Cardim, Lorenzo Ball, Denise Battaglini, Wojciech Dabrowski, Matteo Bassetti, Daniele Roberto Giacobbe, Marek Czosnyka, Rafael Badenes, Paolo Pelosi, Basil Matta, The GeCovid group, Iole Brunetti

**Affiliations:** ^1^Department of Surgical Sciences and Integrated Diagnostics (DISC), University of Genoa, Genoa, Italy; ^2^San Martino Policlinico Hospital, IRCCS for Oncology and Neuroscience, Genoa, Italy; ^3^Department of Neurology, University of Texas Southwestern Medical Center, Dallas, TX, United States; ^4^Department of Medicine, University of Barcelona, Barcelona, Spain; ^5^Department of Anesthesiology and Intensive Care, Medical University of Lublin, Lublin, Poland; ^6^Department of Health Sciences (DISSAL), University of Genoa, Genoa, Italy; ^7^Infectious Diseases Unit, Ospedale Policlinico San Martino, IRCCS for Oncology and Neuroscience, Genoa, Italy; ^8^Brain Physics Laboratory, Department of Clinical Neurosciences, Neurosurgery Unit, Addenbrooke's Hospital, Cambridge, United Kingdom; ^9^Department of Anesthesia and Intensive Care, Hospital Clinic Universitari, INCLIVA Research Health Institute, University of Valencia, Valencia, Spain; ^10^Neurocritical Care Unit, Addenbrooke's Hospital, Cambridge, United Kingdom

**Keywords:** cerebral oxygenation, brain injury, autoregulation dysfunction, intensive care, NIRS (near infrared reflectance spectroscopy)

## Abstract

**Introduction:** The role of near-infrared spectroscopy (NIRS) for the evaluation of cerebral haemodynamics is gaining increasing popularity because of its noninvasive nature. The aim of this study was to evaluate the role of the integral components of regional cerebral oxygenation (rSO_2_) measured by NIRS [i.e., arterial-oxyhemoglobin (O_2_Hbi) and venous-deoxyhemoglobin (HHbi)-components], as indirect surrogates of cerebral blood flow (CBF) in a cohort of critically ill patients with coronavirus disease 2019 (COVID-19). We compared these findings to the gold standard technique for noninvasive CBF assessment, Transcranial Doppler (TCD).

**Methods:** Mechanically ventilated patients with COVID-19 admitted to the Intensive Care Unit (ICU) of Policlinico San Martino Hospital, Genova, Italy, who underwent multimodal neuromonitoring (including NIRS and TCD), were included. rSO_2_ and its components [relative changes in O_2_Hbi, HHbi, and total haemoglobin (cHbi)] were compared with TCD (cerebral blood flow velocity, CBFV). Changes (Δ) in CBFV and rSO_2_, ΔO_2_Hbi, ΔHHbi, and ΔcHbi after systemic arterial blood pressure (MAP) modifications induced by different manoeuvres (e.g., rescue therapies and haemodynamic manipulation) were assessed using mixed-effect linear regression analysis and repeated measures correlation coefficients. All values were normalised as percentage changes from the baseline (Δ%).

**Results:** One hundred and four measurements from 25 patients were included. Significant effects of Δ%MAP on Δ%CBF were observed after rescue manoeuvres for CBFV, ΔcHbi, and ΔO_2_Hbi. The highest correlation was found between ΔCBFV and ΔΔO_2_Hbi (R = 0.88, *p* < 0.0001), and the poorest between ΔCBFV and ΔΔHHbi (R = 0.34, p = 0.002).

**Conclusions:** ΔO_2_Hbi had the highest accuracy to assess CBF changes, reflecting its role as the main component for vasomotor response after changes in MAP. The use of indexes derived from the different components of rSO_2_ can be useful for the bedside evaluation of cerebral haemodynamics in mechanically ventilated patients with COVID-19.

## Introduction

Several neuromonitoring tools have been adopted as surrogate or indirect measures of cerebral blood flow (CBF) (1–3). Among these, transcranial Doppler (TCD)-derived cerebral blood flow velocity (CBFV) has been largely applied, being currently the most used technique for the evaluation of bedside cerebral haemodynamics (4), and is considered as the gold standard for noninvasive assessments of static cerebral autoregulation (CA) (3).

Near-infrared spectroscopy (NIRS) represents a promising but not highly utilised technique in critical care. NIRS measures the relative proportion of oxy (O_2_Hbi)- and -deoxy(HHbi) haemoglobin based on the transmission and absorption of near-infrared light as it passes through biological tissues (5, 6). For instance, NIRS enables the continuous non-invasive evaluation of cerebral oxygenation (6), which could potentially be used as a surrogate of CBF. A major concern of NIRS is the undesired contamination of extracranial blood and the intracranial venous blood (7, 8). However, recent improvement in technology has led to the availability of tools, which can measure not only the total rSO_2_ but also discriminate its different components, e.g., the arterial and venous compartments of cerebral circulation. This could potentially lead to a more detailed evaluation of CBF with NIRS, focusing on the role of different intracerebral components in the vasomotor response, and to assess the effect of mean arterial blood pressure (MAP) changes, specifically considering the arterial component of rSO_2_, thus minimising any confounding factors from the venous compartment. Such assessments can be relevant in acute critical care patients, especially those in risk of developing neurological complications due to non-traumatic brain injuries. In the temporal context of the present work, the severe acute respiratory syndrome coronavirus 2 (SARS-CoV-2) pandemic has led to insurmountable clinical challenges regarding the assessment of such potential, unknown neurological complications that could become another major burden to coronavirus disease 2019 (COVID-19) survivors.

Therefore, the aim of this study was to assess the role of rSO_2_ and its different components as surrogates of CBF in a cohort of mechanically ventilated critically ill patients with COVID-19 undergoing different rescue therapies and haemodynamic manipulations yielding MAP changes. The primary objective was to assess the correlation of the different components of rSO_2_ with the gold standard CBFV. The secondary aim was to investigate which component of rSO_2_ is the best predictor of CBF in response to haemodynamic changes. We hypothesised that the arterial component of rSO_2_ is better correlated with CBFV and should better reflect MAP changes than its venous component, or total rSO_2_, after systemic blood pressure changes.

## Methods

We adhered to the “Strengthening the Reporting of Observational Studies in Epidemiology (STROBE)” statement guidelines for observational cohort studies (Supplementary Material 1) (9).

This single centre prospective, observational study was performed at Policlinico San Martino Hospital, IRCCS for Oncology and Neuroscience, Genoa, Italy, from October 1, to December 15, 2020, and the protocol was approved by the local ethical review board (Comitato Etico Regione Liguria, protocol n. CER Liguria: 23/2020). Written consent was obtained from the next of kin, as the patients were unconscious at the time of inclusion.

Adult mechanically ventilated patients admitted to ICU [previously included in two studies (10, 11) from our group]; with a confirmed SARS-CoV-2 polymerase chain reaction test using nasopharyngeal swab or bronchoalveolar lavage; requiring any type of manoeuvre clinically indicated to optimise respiratory and haemodynamic functions with an effect on ABP; and who contemporarily underwent multimodal neuromonitoring (NIRS and TCD) were included.

These manoeuvres comprised ventilatory rescue therapies (such as recruitment manoeuvres, prone positioning, etc.) or haemodynamic strategies (such as passive leg raising test, fluid challenges, and norepinephrine administration) as previously described (10, 11). Despite different indications and types of manoeuvres, they all had in common to produce an effect on systemic haemodynamics and MAP, which could affect cerebral hemodynamics.

Exclusion criteria were patients negative for SARS-CoV-2 infection; patients who did not undergo any intervention with a haemodynamic effect, or who did not receive multimodal neuromonitoring (TCD and NIRS) or with known neurological conditions before or during ICU admission, which might have impaired cerebral haemodynamics *a priori* (stroke, trauma, intracerebral masses, etc.).

### Patient Management and Data Collection

A combination of propofol, midazolam, and fentanyl was used to maintain sedation. The patients were mechanically ventilated using pressure-controlled ventilation, using a tidal volume (V_T_) of 4–8 ml/kg of predicted body weight, aiming to achieve plateau pressure (Pplat) <28 cmH_2_0; inspired fraction of oxygen (FiO_2_) and positive end expiratory pressure (PEEP) were titrated to achieve peripheral saturation of oxygen (SpO_2_) = 88–92%, and the respiratory rate was set to maintain arterial partial pressure of carbon dioxide (PaCO_2_) = 35–45 mmHg. Indications and methods for each one of the strategies considered for the analyses have been previously presented in detail (10).

Data were reviewed and collected by physicians trained in critical care (10). Data collection included baseline characteristics, demographics, clinical data at ICU admission, laboratory and ventilatory parameters (i.e., V_T_, FiO_2_, PEEP), as well as haemodynamic and neuromonitoring data before (T0) and after (T1) the application of any type of manoeuvre. In brief, T1 measurements were taken after 5 min after recruitment manoeuvres and prone positioning, 5 min after the beginning of norepinephrine administration and fluid challenges administration, and immediately after passive leg raising test (10, 11). These procedures are standardised in our institution and, therefore, were expected to obtain a similar haemodynamic effect.

The decision to start any type of manoeuvre was related to the assessment and judgement of the clinician. Details on the different manoeuvres have been previously described (10, 11).

### Neuromonitoring

#### Cerebral Oxygenation

We used the Masimo Root monitor® (Irvine, CA, USA) for the measurement of rSO_2_, applying bilateral sensors to the frontotemporal area of the patients. Final values of rSO_2_ and its components at T0 and T1 were calculated as the mean between single instant measurements obtained from the right and left frontotemporal sensors.

The parameters obtained included:

- rSO_2_, which represents the total regional cerebral oxygen saturation;
- ΔO_2_Hbi, which represents the change in the oxyhemoglobin component of the rSO_2_ calculation, i.e., changes in the cerebral arterial compartment;
- ΔHHbi, which represents the change in the deoxyhemoglobin component of the rSO_2_ calculation, i.e., changes in the cerebral venous compartment;
- ΔcHbi, the sum of the values of ΔO_2_Hbi e ΔHHbi.

#### Transcranial Doppler

A low frequency (2 MHz) echographic micro convex probe was employed to investigate intracranial vessels. The temporal window was privileged for the passage of Doppler signals for middle cerebral artery (MCA) insonation. Single instant measurements of mean cerebral blood flow velocities (CBFV) were obtained bilaterally from the MCA at T0 and T1. Cerebrovascular resistance (CVRi - pressure gradient/flow) and conductance (CVCi - flow/pressure gradient) indices of vasoconstriction and vasodilation were also calculated at each time point.

### Statistical Analysis

The Shapiro–Wilk test was used to test the normality of the distribution of the results. Data are reported as the median and interquartile range [IQR = 25th−75th percentiles]. Comparisons between different variables at T0 and T1 were performed by the Wilcoxon-signed rank test.

Dependent variables were expressed as a change from the baseline (T0) in both absolute (Δ change) and relative terms (Δ% change). Changes in CBFV, NIRS parameters, CVCi, and CVRi (both derived from the ratio of mean CBFV/NIRS parameters and MAP) from the baseline were used to show changes for these dependent variables across the range of MAP in response to the rescue manoeuvres.

The correlations coefficients [95% confidence interval (CI)] between CBFV and the different NIRS variables were verified using the Bland and Altman method for repeated measurements (12, 13).

To provide prediction models for the relationship between CBFV and NIRS parameters with the observed changes in MAP, the relationships between Δ% CBFV and the Δ% NIRS parameters (rSO_2_, ΔO_2_Hbi, ΔHHbi, and ΔcHbi) with Δ% MAP were expressed as linear mixed effects models [*R* Software's package *lme4* (14)]. As fixed effects, we entered Δ% CBFV or Δ% NIRS parameters and Δ% MAP into each respective model. As random effects, we had intercepts and slopes for the repeated measurement points for each patient (*N* = 104 measurements).

All statistical analyses were performed using RStudio software (version 4.0.3). A *p* < 0.05 was considered statistically significant.

## Results

One hundred and four measurements from 25 patients were analyzed. The patients had a range of three to six manoeuvres performed (mean ± SD: 4.16 ± 0.88). The characteristics of the patients are presented in Supplementary Table S1. The median age was 62 [57–69], and 80% were male. Fifteen patients (60%) died in ICU, and five (20%) patients were diagnosed with delirium. Non-survivors had smaller changes in CBFV and O_2_Hbi compared to survivors (−5 vs. 2 cm/s, *p* < 0.001, and −1.5 vs. 0.25-μM cm, *p* = 0.02, respectively).

Main variables at T0 and T1 are described in Table 1. At T1, compared to T0, MAP significantly increased by 6.0 (−1.25–8.25) mmHg (*p* < 0.0001); CBFV significantly decreased by −4.0 [−7–(−2)] cm/s (*p* = 0.03); ΔO_2_Hbi decreased by −1.3 (−1.9-0.3) (μM.cm) (*p* < 0.001); ΔHHbi significantly increased by 0.9 (0.1–1.3) (μM.cm) (*p* < 0.0001). PaCO_2_ decreased by −1.0 [−5.25–3.0] mmHg, although not statistically significant (*p* = 0.06). SpO_2_ significantly increased by 6.0 (−0.0–29.2) % (*p* < 0.0001).

**Table 1:** Systemic and brain haemodynamics haemodynamics and oxygenation at baseline (T0) and post-event (T1). Data are presented as median (IQR).

	**MAP (mmHg)**	**CBFV (cm/sec)**	**rSO_**2**_ (%)**	**ΔcHbi**	**ΔO_**2**_Hbi**	**ΔHHbi**	**PaCO_**2**_ (mmHg)**	**SpO_**2**_ (%)**
**T0**	65.0 (61.0–71.0)	56.0 (50.0–59.2)	53.0 (51.7–58.0)	4.7 (3.2–6.3)	3.5 (2.8–4.4)	1.1 (0.4–1.9)	51.0 (46.0–66.2)	86.5 (61.0–89.0)
**T1**	69.5 (66.0–73.2)	53.0 (46.0–58.2)	57.0 (53.0–59.0)	4.8 (3.3–7.0)	2.7 (1.6–4.2)	1.9 (1.3–2.8)	48.0 (45.0–56.0)	91.0 (89.0–92.0)
**Δ** **changes**	**6.0 (−1.2–8.2)**	**−4.0 (−7–(−2))**	2.0 (−3.2–5.2)	−0.2 (−1.7–1.3)	**−1.3 (−1.9–0.3)**	**0.9 (0.1–1.3)**	−1.0 (−5.2–3.0)	**6.0 (0.0–29.2)**

*CBFV, cerebral blood flow velocity (cm/s); ΔcHbi, sum of ΔO_2_Hbi and ΔHHbi components of the regional tissue oxygen saturation (μM.cm); ΔO_2_Hbi, index representing the change in the oxyhemoglobin of the regional tissue oxygen saturation (μM.cm); ΔHHbi, index representing the change in the deoxyhemoglobin of the regional tissue oxygen saturation (μM.cm); rSO_2_, regional tissue oxygen saturation (%); MAP, mean arterial blood pressure (mm Hg); PaCO_2_, partial pressure of CO_2_ (mm Hg); SpO_2_, systemic oxygen saturation (%). Bold values indicate statistical significance (p < 0.05)*.

**Figure 1:**
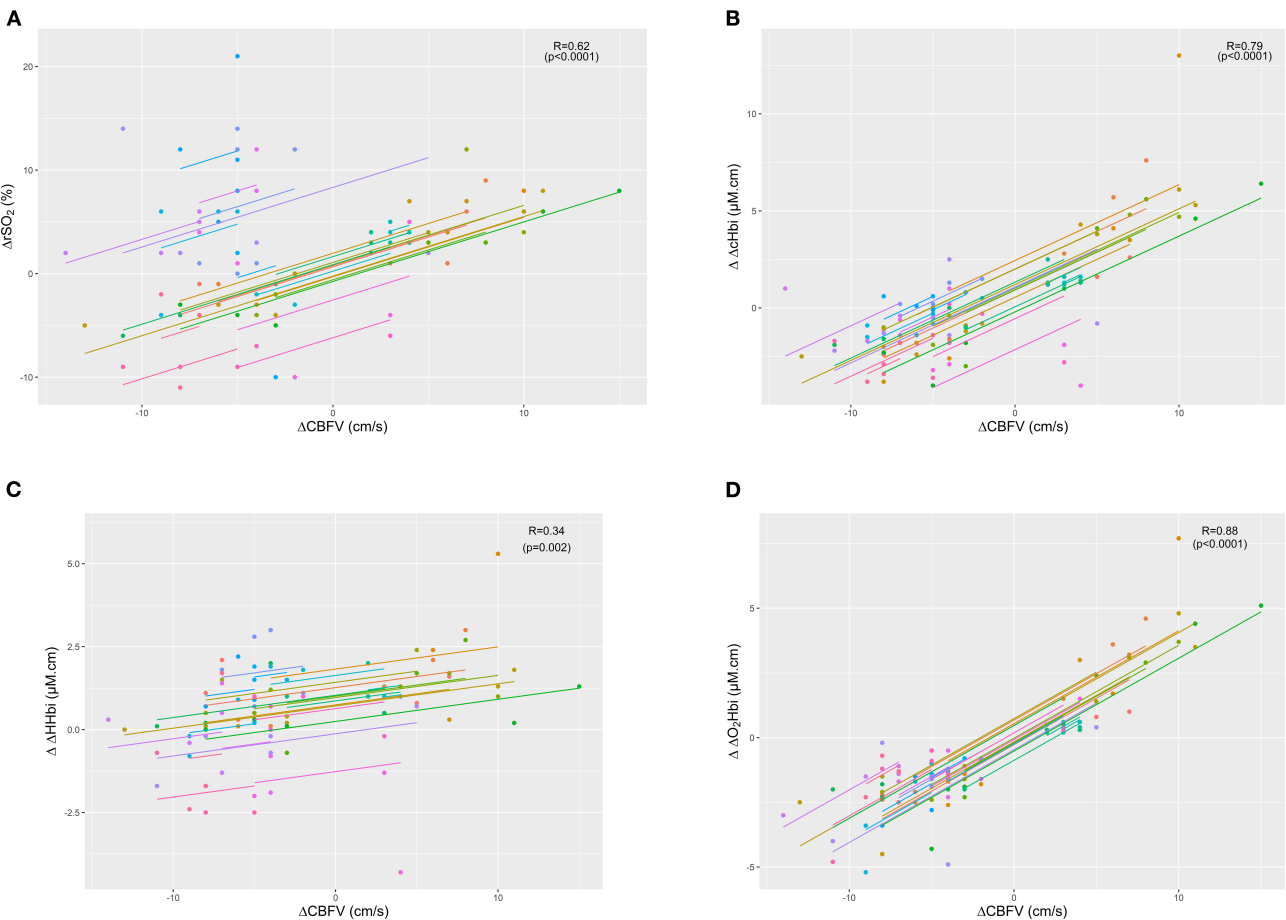
Scatter plots showing the linear association and correlation (R) between changes in cerebral blood flow velocity (ΔCBFV) vs. total cerebral oxygenation (ΔrSO_2_ - plot A) **(A)**, sum of arterial and venous components of cerebral oxygenation (ΔΔcHbi - plot B) **(B)**, venous component (ΔΔHHbi - plot C) **(C)**, and arterial component (ΔΔO_2_Hbi - plot D) **(D)**. Repeated measurements for each patient are plotted in the same colour pattern. Linear regression lines are correspondent to repeated measurements within patients.

ΔCBFV, the gold standard for CBF assessment, showed the highest correlation with the arterial component of cerebral oxygenation (ΔO_2_Hbi; R = 0.88 (0.81–0.92), *p* < 0.0001), followed by ΔcHbi and ΔrSO_2_ values (R = 0.79 (0.68–0.86), *p* < 0.0001 and R = 0.62 (0.46–0.74), *p* < 0.0001) (Figure 1). The lowest correlation was found for ΔHHbi [R = 0.34 (0.13–0.52), *p* = 0.002]. Table 2 shows the correlation coefficients of cerebrovascular resistance index (CVRi) and cerebrovascular conductance index (CVCi) for CBFV and NIRS parameters. Consistently, NIRS parameters of CVCi, not CVRi, were better correlated with CBFV.

**Table 2:** Correlation of cerebrovascular resistance index (CVRi) and cerebrovascular conductance index (CVCi) for cerebral blood flow velocity (CBFV) and NIRS parameters.

	**ΔCVRi_**CBFV**_**	***p*–value**		**ΔCVCi_**CBFV**_**	***p*–value**
**ΔCVRi**_**rSO2**_	0.99 (0.99–0.99)	**<0.0001**	**ΔCVCi**_**rSO2**_	0.8 (0.71–0.81)	**<0.0001**
**ΔCVRi**_**Δ**_ **cHbi**	0.17 (−0.05–0.38)	0.12	**ΔCVCi**_**Δ**_ **cHbi**	0.62 (0.47–0.74)	**<0.0001**
**ΔCVRi**_**Δ**_ **O2Hbi**	0.28 (0.06–0.47)	**0.01**	**ΔCVCi**_**Δ**_ **O2Hbi**	0.71 (0.58–0.80)	**<0.0001**
**ΔCVRi**_**Δ**_ **HHbi**	0.01 (−0.22–0.23)	0.95	**ΔCVCi**_**Δ**_ **HHbi**	0.32 (0.11–0.51)	**0.004**

*CBFV, cerebral blood flow velocity; CI, confidence interval; ΔcHbi, sum of ΔO_2_Hbi and ΔHHbi components of the regional tissue oxygen saturation; ΔO_2_Hbi, index representing the change in the oxyhemoglobin of the regional tissue oxygen saturation; ΔHHbi, index representing the change in the deoxyhemoglobin of the regional tissue oxygen saturation; rSO_2_, regional cerebal oxygenation*.

**Figure 2:**
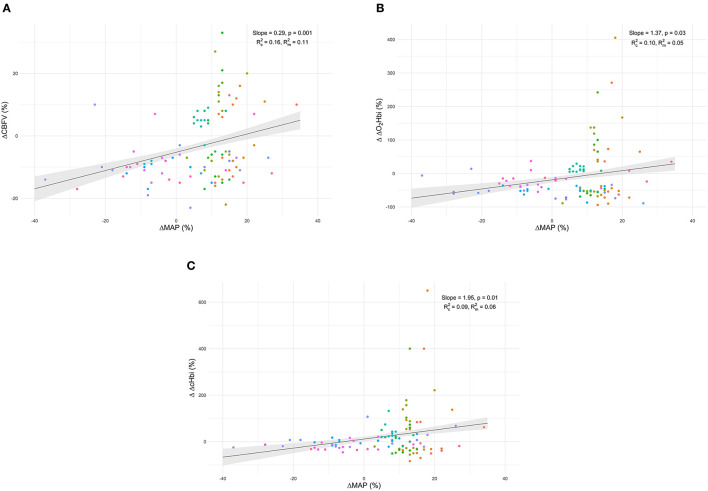
Individual data of cerebral blood flow relative response changes to MAP from T0 to T1 and linear mixed-effects models marginal mean values of CBF for cerebral blood flow velocity (ΔCBFV–plot A) **(A)**, the arterial component of cerebral oxygenation (ΔO_2_Hbi–plot B) **(B)**, and arterial plus venous components (ΔcHbi–plot C) **(C)**.

Individual data of CBF responses to changes in MAP from T0 to T1 and linear mixed-effects models marginal mean values of CBF are presented in Figure 2. Model parameters for the fixed effects of the Δ% CBF to Δ% MAP models are presented in Table 3. Significant effects of Δ% MAP on Δ% CBF from T0 to T1 were observed for CBFV (*p* = 0.001) and for ΔcHbi and ΔO_2_Hbi (*p* = 0.01 and 0.03, respectively) (Figures 2A–C, Table 3), but not for ΔHHbi and rSO_2_. Inclusion of PaCO_2_ as a covariate did not improve any of the models fits. Boxplot panels showing the delta difference (T1-T0) for each patient concerning the different parameters analysed are shown in ESM Supplementary Figure S1. A representative patient, showing the individual trajectories of the different parameters across the measurement points, is shown in ESM Supplementary Figure S2.

**Table 3:** Linear mixed–model estimates of fixed effects of CBF responses to changes in MAP.

	**Models**	**Parameter**	**Estimate**	**Std. Error**	**p value**	**95% Confidence Interval**	
						**Lower bound**	**Upper bound**
Δ% CBFV	Model 1	β*_0_ Intercept*	−5.24	1.19	**<0.001**	−7.76	−2.68
		β*_1_* Δ% *MAP*	0.29	0.08	**0.001**	0.11	0.50
	**AIC**	800.2					
	${\text{R}}_{\text{m}}^{2}$	0.11					
	${\text{R}}_{\text{c}}^{2}$	0.16					
Δ% ΔcHbi	Model 2	β*_0_ Intercept*	0.11	0.10	0.28	−0.10	0.32
		β*_1_* Δ% *MAP*	1.95	0.77	**0.01**	0.42	3.58
	**AIC**	296.8					
	${\text{R}}_{\text{m}}^{2}$	0.06					
	${\text{R}}_{\text{c}}^{2}$	0.09					
Δ% ΔO_2_Hbi	Model 3	β_0_ *Intercept*	−0.19	0.08	**0.02**	−0.36	−0.02
		β*_1_* Δ% *MAP*	1.37	0.62	**0.03**	0.12	2.80
	**AIC**	247.3					
	${\text{R}}_{\text{m}}^{2}$	0.05					
	${\text{R}}_{\text{c}}^{2}$	0.10					

*AIC, Akaike information criterion; CBFV, cerebral blood flow velocity; ΔcHbi, sum of ΔO_2_Hbi and ΔHHbi components of the regional tissue oxygen saturation; ΔO_2_Hbi, index representing the change in the oxyhemoglobin of the regional tissue oxygen saturation. $R_{m}^{2}$: marginal coefficient of determination of the model, describing the proportion of variance explained by the fixed effects. $R_{c}^{2}$: conditional coefficient of determination of the model, describing the proportion of variance explained by both the fixed and random effects*.

## Discussion

In a cohort of mechanically ventilated patients with COVID-19 undergoing different rescue therapies and haemodynamic manipulations, we found that: (1) ΔO_2_Hbi was better correlated with ΔCBFV than ΔHHbi and other components of cerebral oxygenation. ΔHHbi showed the poorest correlation with ΔCBFV; (2) CVCi indices derived from NIRS parameters, especially rSO_2_, correlated consistently better with CBFV than CVRi; (3) changes in MAP led to cerebral vasoreactive responses, which could be detected using the arterial component of rSO_2_, but not total rSO_2_ or its venous component. Our data suggest that, as the arterial compartment is responsible for the major changes in cerebrovascular tone following arterial blood pressure changes, the ΔO_2_Hbi component of NIRS can more specifically describe these variations.

To our knowledge, this is the first study evaluating the role of NIRS in response to secondary changes in systemic haemodynamics in mechanically ventilated patients with COVID-19. Compared to our previous work (11), where CA was measured with TCD, we aimed to use for the first time NIRS and its components for the assessment of CBF changes, comparing it with TCD, the gold standard for noninvasive cerebral haemodynamics assessment. The novelty of this study relies on the use and comparison of the different components of rSO_2_ to assess CBF, which is allowed by the Masimo® device. We believe that this is important, as there is a knowledge gap concerning the comparison of specific components of the NIRS signal with CBFV.

Although NIRS has been previously used as surrogate of cerebral blood flow in the dynamic measurement of autoregulation (15, 16), its use is importantly limited by the fact that it takes into account both arterial and venous cerebral compartments, which do not take part in the same way for CBF regulation. Moreover, it has been mainly used in the paediatric population (15, 16), and data on patients with COVID-19 are lacking.

Although this is just a preliminary study, our results suggest that using only the arterial component of rSO_2_ could improve the accuracy in estimating CBF changes, eliminating the venous contamination (17). Furthermore, NIRS can provide reliable, practical, and quick assessments of cerebral haemodynamics in the challenging setting of critical care in the patients with COVID-19 with acute respiratory syndrome, in whom the development of neurological complications are common (18).

Several studies have highlighted the importance of the evaluation of cerebral haemodynamics and CA in the management of patients who are brain injured and patients who are non-brain injured (3–5). At present, no randomised controlled trials exploring the effect on the outcome of a therapeutic approach based on CA are available (19, 20); however, experts agree that the maintenance of the homeostasis of autoregulatory status of patients should be kept in consideration when managing patients who are brain injured, as impaired CBF dynamics can lead to secondary cerebral insult (6, 19).

The Brain Trauma Foundation Guidelines only suggest to maintain a cerebral perfusion pressure (CPP) of 60–70 mmHg in patients who are traumatically brain injured (21). However, the use of one single target of CPP for all the patients may not represent the individual physiological needs and CA changes over time (22, 23). An individualised management of CBF, based on the intrinsic autoregulatory state of the single patients and its changes over time, may better represent the physiological changes occurring in the vasomotor response of patients with brain injury (3, 24–26).

Amongst the methods proposed as possible surrogates of CBF (24), neuroimaging techniques, such as Positron Emission Tomography (PET) and Single-Photon Emission Computed Tomography (SPECT), are very accurate, but these are expensive, time-consuming, not available at bedsides of patients, and have a limited role in the clinical context (24).

A number of indirect techniques have been proposed based on invasive surrogates of CBF (intracranial pressure, invasive cerebral oxygenation monitoring) and non-invasive methods (TCD, NIRS) (24). Optical techniques, which can directly measure flow (i.e., diffuse correlation spectroscopy), and dynamic contrast enhanced NIRS have been used to measure CBF and validated against PET for regional cerebral blood flow measurement (25), but there is a paucity of studies in the literature regarding their use to assess cerebral haemodynamic responses (26).

CBF and CA can be evaluated by measuring relative blood flow changes in response to a steady-state change in the blood pressure (static methods) or during the response to spontaneous changes in blood pressure (dynamic methods) (23). Although dynamic methods do not require MAP manipulations (which can be dangerous in patients who are critically ill) and better reflect the changes of CA over time, they require the use of software, which is not always available at bedsides of patients (24). After MAP increase, a contemporary augmentation of CBFV, for instance, indicates a loss of CA, whereas, if CBFV decreases or remains constant, it indicates a functioning vasomotor response and preserved CA (27).

Outside the assessment of CA using TCD-based indices obtained *via* neuromonitoring software platforms (limited to few centres with technical resources and relevant clinical expertise), the simple assessment of CVRi/CVCi in response to systemic stimuli can be performed. Although routine assessment of these metrics is not always feasible, they present wide clinical applications, from the determination of stroke risk in individuals with severe asymptomatic carotid or intracranial steno-occlusive disease to concussions and patients with neurodegenerative conditions, such as Alzheimer's disease. In fact, previous works have described that changes in conductance (CVCi) better reflect the response in pressure regulation than do changes in resistance (CVRi) (28). This hypothesis is confirmed by the present data showing that CVCi measured by rSO_2_ correlated consistently better with CBFV when compared to CVRi in response to rescue therapies and haemodynamic changes.

In this context, rSO_2_ could potentially be applied as surrogate of CBF, as NIRS- similarly to TCD- is a low cost, easy available, and safe neuromonitoring tool (27). For example, NIRS has been previously used for the dynamic assessment of CA, with promising results, but several concerns have been raised regarding its routine use (7, 8, 29, 30). In particular, as NIR light penetrates skin, subcutaneous fat, skull, and underlying muscles and brain tissue, the absorption of light from chromophores depends on both the changes in oxygenated and deoxygenated haemoglobin, and, therefore, from both the arteriolar and venular beds. As a consequence, it has been shown that the absolute number of rSO_2_, as well as its changes, might not be always reliable in practise since venous contamination can lead to misinterpretation of the data (29, 31).

The use of a device capable of effectively discriminating the changes of the arterial and venous compartments can, therefore, improve the accuracy of NIRS in different clinical contexts, especially in the assessment of CBF. As CBF is mainly regulated by the vasomotor response, which is a mechanism that primarily affects the arterial compartment, we have demonstrated that, by eliminating the effect of the venular bed, the estimation of CBF is more accurate than using the total rSO_2_ values. Therefore, we believe that our results could pave the way for the application of a new technique for a quick and bedside assessment of CBF, with the adoption of tools, which are currently easily available and can provide important bedside information for cerebral haemodynamics assessment and management.

There are several limitations in this study that need to be mentioned. Firstly, although TCD is considered the gold standard for evaluation of cerebral haemodynamics, it only represents an indirect surrogate of CBF. A linear relationship has been demonstrated between CBFV and CBF (3), but with the assumption that the diameter of the insonated artery remains constant over time. Secondly, although in this study we included all rescue manoeuvres with a potential effect on MAP, these included a heterogeneous number of interventions (including fluids and norepinephrine administration, prone positioning, recruitment manoeuvres etc.); therefore, the increase in MAP was not standardised in the measurements. Typically, static cerebral autoregulation is assessed when MAP is increased by continuous infusion of a vasopressor (such as phenylephrine) to slowly increase MAP by ~20 mm Hg. In our study, MAP increased only by 6 mm Hg. As this was an observational study, we could not target to a specific MAP to guide our treatment; we just observed the effect of a treatment on different patients. This is an important limitation as, in our cohort, we were able to assess mainly the changes of CBF rather than static CA. In fact, as previously reported, the accuracy of estimating the autoregulatory curve, or its different segments, from only two points is considered unreliable (32).

In addition, TCD evaluates major arteries, while NIRS measures the cerebral oxygenation at the microvasculature level (i.e., tissue oxygenation). This could lead to a decoupling between large and micro vessels. However, both methods are currently considered as surrogate of CBF (24, 33). Finally, we did not have measurements of the arteriovenous oxygen content difference concomitantly with the TCD/NIRS assessments to determine the cerebral metabolic rate of oxygen.

## Conclusions

The arterial component of rSO_2_ had the highest accuracy in assessing CBF changes, reflecting its role as the main component for vasomotor response after changes in MAP. These findings indicate that CBF assessed with NIRS, specifically through ΔO_2_Hbi, is comparable to TCD in this patient population. The use of indices derived from the different components of rSO_2_ can be useful for the bedside evaluation of cerebral haemodynamics in patients who are critically ill in order to optimise haemodynamics of patients and potentially improve their outcome. Further studies are warranted to better define the role of this technique in the clinical practise, especially the continuous assessment of cerebral autoregulation in mechanically ventilated patients with COVID-19, with acute respiratory syndrome.

## The Gecovid Group

Ospedale Policlinico San Martino, IRCCS for Oncology and Neuroscience, Genoa, Italy: Iole Brunetti, Maurizio Loconte, Fabio Tarantino, Marco Sottano, Francesco Marramao, Angelo Gratarola, Paolo Frisoni. Department of Surgical Sciences and Integrated Diagnostics (DISC), University of Genoa, Italy: Elena Ciaravolo. Infectious Diseases Unit, Ospedale Policlinico San Martino, Genoa, Italy: Chiara Dentone, Lucia Taramasso, Laura Magnasco, Antonio Vena. Department of Neurosurgery, Ospedale Policlinico San Martino, Genoa, Italy: Gianluigi Zona. Department of Neurosurgery, Ospedale Policlinico San Martino, Genoa, Italy: Pietro Fiaschi.

## Data Availability Statement

The raw data supporting the conclusions of this article will be made available by the authors, without undue reservation.

## Ethics Statement

The studies involving human participants were reviewed and approved by Comitato Etico Regione Liguria, protocol n. CER Liguria: 23/2020. The patients/participants provided their written informed consent to participate in this study.

## Author Contributions

CR writing of the manuscript, paper revision, fundamental conceptual contribution, and data analysis. LB, DB, DG, WD, MB, MC, RB, PP, and BM paper revision and fundamental conceptual contribution. DC, paper revision, fundamental conceptual contribution, and data analysis. All authors contributed to the article and approved the submitted version.

## Conflict of Interest

BM is the senior medical director of Masimo. CR, PP, and RB report personal fees from Masimo. MB reports personal fees and others from Angelini, personal fees and others from AstraZeneca, others from Bayer, personal fees and others from Cubist, personal fees and others from Pfizer, personal fees and others from Menarini, personal fees and others from MSD, others from Nabriva, others from Paratek, others from Roche, others from Shionogi, others from Tetraphase, others from the Medicine Company, personal fees and others from AstellasPharma Inc., personal fees from Gilead Sciences, personal fees from Teva, personal fees from Novartis, grants from Ranbaxy, personal fees from Correvio, personal fees from Molteni, personal fees from Thermo Fisher, outside the submitted work. Dr Herrmann is a cofounder and shareholder in OscillaVent, Inc, and a consultant for ZOLL Medical Corporation, both outside the submitted work. MC is recipient of part of the licensing fee for ICM+ software (Cambridge Enterprise Ltd., UK) outside the submitted work. DG reports personal fees from Stepstone Pharma GmbH, personal fees from MSD Italia and personal fees from Correvio Italia, outside the submitted work. The remaining authors declare that the research was conducted in the absence of any commercial or financial relationships that could be construed as a potential conflict of interest.

## Publisher's Note

All claims expressed in this article are solely those of the authors and do not necessarily represent those of their affiliated organizations, or those of the publisher, the editors and the reviewers. Any product that may be evaluated in this article, or claim that may be made by its manufacturer, is not guaranteed or endorsed by the publisher.
